# The Emerging Role of the Major Histocompatibility Complex Class I in Amyotrophic Lateral Sclerosis

**DOI:** 10.3390/ijms18112298

**Published:** 2017-11-01

**Authors:** Gabriela Bortolança Chiarotto, Giovanni Nardo, Maria Chiara Trolese, Marcondes Cavalcante França, Caterina Bendotti, Alexandre Leite Rodrigues de Oliveira

**Affiliations:** 1 Department of Structural and Functional Biology, Institute of Biology—Unicamp, 13083-865 Campinas, Brazil; gabrielachiarotto@gmail.com; 2 Department of Neuroscience, IRCCS—Institute of Pharmacological Research Mario Negri, 20156 Milano, Italy; giovanni.nardo@marionegri.it (G.N.); mariachiara.trolese@marionegri.it (M.C.T.); 3 Departament of Neurology, Faculty of Medical Sciences—Unicamp, 13083-887 Campinas, Brazil; mcfrancajr@uol.com.br

**Keywords:** major histocompatibility complex I, amyotrophic lateral sclerosis, glial cells, microglia, neuroprotection

## Abstract

Amyotrophic lateral sclerosis (ALS) is a fatal neurodegenerative disease affecting upper and lower motoneurons (MNs). The etiology of the disease is still unknown for most patients with sporadic ALS, while in 5–10% of the familial cases, several gene mutations have been linked to the disease. Mutations in the gene encoding Cu, Zn superoxide dismutase (SOD1), reproducing in animal models a pathological scenario similar to that found in ALS patients, have allowed for the identification of mechanisms relevant to the ALS pathogenesis. Among them, neuroinflammation mediated by glial cells and systemic immune activation play a key role in the progression of the disease, through mechanisms that can be either neuroprotective or neurodetrimental depending on the type of cells and the MN compartment involved. In this review, we will examine and discuss the involvement of major histocompatibility complex class I (MHCI) in ALS concerning its function in the adaptive immunity and its role in modulating the neural plasticity in the central and peripheral nervous system. The evidence indicates that the overexpression of MHCI into MNs protect them from astrocytes’ toxicity in the central nervous system (CNS) and promote the removal of degenerating motor axons accelerating collateral reinnervation of muscles.

## 1. Introduction

Neurodegenerative diseases are devastating conditions that result in a significant loss of function and quality of life. Among them, amyotrophic lateral sclerosis (ALS) is the most prevalent motoneuron (MN) disease in adults, specifically targeting MNs in the cerebral cortex, brainstem, and spinal cord, causing progressive denervation and the atrophy of skeletal muscles, which finally results in complete paralysis and death [1].

Symptoms and signs emerge when axonal connections fail, that is, when the axon retracts and denervation of the lower MNs or the muscle occurs. In fact, terminal axonal degeneration and neuromuscular junction (NMJ) denervation occur well before the loss of MNs in mutant superoxide dismutase (mSOD1) mice and ALS patients [2].

ALS was first described by the French neurologist Jean-Martin Charcot in 1869, as a neurodegenerative disease that begins in late adulthood. Nowadays, it is considered the fourth most common neurodegenerative cause of death in adults after Alzheimer’s, Parkinson’s, and Huntington’s disease with a prevalence of 4–8 per 100,000 individuals [1,3]. The average patient survival is 2–5 years from the onset of symptoms, although about 10% survive longer than 10 years [4,5]. The diagnosis essentially relies upon the exclusion of other similar neuromuscular diseases.

ALS is a complex disease that can be divided into two forms, namely sporadic and familial. The most common form of ALS is sporadic (sALS), whereas the dominantly inherited familial ALS (fALS) associated with mutations in more than 40 genes accounts for only about 5–10% of all affected patients [6,7]. Of these, mutations in the SOD1 gene, which account for 20% of fALS and 5% of sALS, have been the most investigated [8]. More recently, hexanucleotide (GGGGCC) expansions in the first intron of the *C9orf72* gene were recognized as the most frequent cause of fALS. They were found in about 40% of fALS and 10% of sALS [9]. Mutations in other genes, including TARDBP [10] and FUS/TLS [11], are together responsible for only 10% of familial cases and about 4–9% of sporadic ALS [9].

Over the years, TDP43, FUS, and C9ORF72 animal models have been generated [12,13]. However, transgenic rodent SOD1 mutants remain, to date, the most widely investigated model that best recapitulates different key features of ALS. These animals are useful for deciphering cellular and molecular disease mechanisms and for testing the potential efficacy of novel therapeutic interventions [8].

The exact mechanisms whereby SOD1 mutants make the MNs highly vulnerable and contribute to disease progression is not yet completely defined. However, in the last years, in vivo and in vitro studies, using transgenic mice carrying SOD1 mutations, demonstrated that the degeneration of MNs was driven by acquired toxicities of mutant protein rather than from reduced dismutase activity [14]. A prominent finding is that mutant SOD1 (mSOD1) fails to fold properly, leading to an accumulation of the misfolded protein, which contributes to the toxicity in ALS. This phenomenon is not exclusive to mSOD1, as other ALS-related genes such as *TARDBP* are associated with an accumulation of ubiquitinated TDP43 aggregates in MNs for almost all ALS patients [7]. Protein misfolding and aggregation may also arise from the aberrant oxidation of intracellular proteins, i.e., increased nitration of tyrosine residues, as a consequence of oxidative stress induced either by mutant SOD1 or other pathogenic mechanisms [15,16,17,18]. In this scenario, mitochondrial dysfunction detected in ALS models and patients plays an important role in the process of ROS accumulation and the subsequent generation of oxidative stress leading to irreversible cell damage [19,20]. In fact, mitochondrial derangement, as well as an increase in markers of oxidative stress and reactive oxygen species (ROS) e.g., 3-nitrotyrosine, have been reported not only in mSOD1 mouse models but also in skeletal muscle biopsies [21] and post-mortem tissues from sporadic ALS patients [22]. Increased nitration of tyrosine residues alter the function of important proteins and/or promote their degradation leading to cell damage [23]. However, protein degradation systems such as the ubiquitin-proteasome pathway, the autophagy, and the endoplasmic reticulum (ER)-associated degradation (ERAD) pathway are impaired in the MNs of ALS animal models, an obvious consequence of which is the accumulation of misfolded protein and the formation of ubiquitinated inclusions, that are typical hallmarks in ALS [24,25,26,27,28].

Other potential pathogenic mechanisms have been associated with the vulnerability of MNs, and the important role of neuroinflammation mediated by glial and immune cells that are active player in the pathogenesis of ALS is emerging [14]. In fact, it is now clear that MN vulnerability is not a cell-autonomous mechanism. As such, other cell types within CNS and periphery, including microglia, astrocytes, oligodendrocytes, macrophages, and T cells, contribute to MN injury [29,30,31]. For instance, while the expression of mSOD1 in cultured MNs alone is not sufficient to trigger their degeneration [32], the presence of mutant SOD1 astrocytes and/or microglia induce significant MN loss due to released neurotoxic factors [33,34,35]. Consistently, ablation of mSOD1 in astrocytes and microglial cells increase the MN survival and lifespan of mSOD1 transgenic mice [33,36,37]. Additionally, peripheral immune cells, like macrophages and T cells, are actively involved in pathogenesis and progression, but their role is still controversial. In fact, while an increased recruitment of inflammatory monocytes/macrophages has been suggested to play a detrimental role in disease progression [38], deficiency in T cells led to an accelerated disease progression of mutant SOD1 transgenic mice [39,40]. This complies with growing evidence that both local inflammatory cells and peripheral immune system mediate either a protective or deleterious effect on MN survival and these functions may vary during disease progression.

Although these observations point to the active role of non-neuronal cells in the development and progression of the pathology, the source(s) and interaction of these pathogenic processes leading to MN death in ALS remain largely unknown [41]. Understanding the complexity of these mechanisms is crucial for developing therapies designed to delay or even prevent the degeneration of MNs in ALS.

It is becoming evident that MNs express an unusual number of molecules that were originally thought to be specific to immune functions [42,43,44]. Some of these molecules, while maintaining the same structure, may also have a different function depending on the context in which they act, a feature termed pleiotropy [45]. One such molecule, for example, is the major histocompatibility complex class I (MHCI), a key molecule of the immune system for the antigen presentation to CD8^+^ T cells, which also has an important function in the activity-dependent refinement and plasticity of connections in developing and adult CNS [46,47,48,49].

## 2. Amyotrophic Lateral Sclerosis (ALS): The Role of the Major Histocompatibility Complex Class I in Motoneurons

Major histocompatibility complex I (MHCI), originally described as a molecule of the immune system, playing an important role in the presentation of antigens, has also become known for the non-immune functions in the nervous system [48,49]. MHCI expression was found in neuronal cells of several regions of CNS in mice including cerebral cortex, hippocampus, striatum, and cerebellum [50,51,52], where it plays a key role in brain development and plasticity [47,53,54,55]. From these studies, it was found that MHCI is required for the establishment of appropriate connections between neurons. This activity is apparently immune-independent and specifically related to the regulation of long-term plasticity of excitatory synaptic transmission [50]. For example, in the absence of MHCI molecules, a surplus of connections between retina and lateral geniculate nucleus is observed [46]. However, when MHCI expression is selectively restored in neurons, synapse pruning in visual areas is re-established [56]. In vitro studies with hippocampal neurons demonstrated that MHCI is important for neurite outgrowth control and cell polarization, whereas neurons overexpressing MHCI generated more primary neurites, presented faster neurite elongation, and accelerated polarization [57].

In the spinal cord, Oliveira et al. [49] showed that the lack of a functional MHCI in β2m^−/−^ mice subjected to axonal transection strongly influenced pre-synaptic density and axonal regeneration of spinal alpha MNs. In fact, in β2m^−/−^ mice, pre-synaptic detachments of lumbar MNs somata one week after peripheral sciatic nerve transection was more extensive than those in wild-type animals. This removal selectively involved clusters of inhibitory synapses without affecting the loss of excitatory synapses that remained the same between normal and β2m^−/−^ mice. These results demonstrate that, after peripheral axon lesion, MNs preferentially remove excitatory inputs, maintaining a subset of inhibitory inputs on their somata, and this last effect is dependent on the presence of MHCI. Additionally, in the same study, Oliveira et al. [49] demonstrated that the number of MNs able to regenerate new axons in the distal nerve stump after axotomy were lower in β2m^−/−^ mice compared to wild-type mice, suggesting a positive effect of MHCI in axonal regeneration. This was subsequently confirmed by other studies showing that wild-type mouse strains with a greater ability to upregulate neuronal MHCI expression after injury had more efficient axonal regrowth [58]. Thus, transgenic mice with enhanced neuronal MHCI expression (NSE-Db) recover locomotor function better after spinal cord injury compared to wild-type mice [59].

Studies in axotomized mice revealed that, while lesioned MNs overexpress MHCI mRNA subunits, the level of the protein was barely detectable in MN somata. On the contrary, a clear accumulation of MHCI was observed in motor axons and terminals at NMJs in gastrocnemius muscles. Consistently, mice defective for MHCI heavy chains (Kb^−^/^−^; Db^−^/^−^ mice), after sciatic nerve injury, showed an abnormal pattern of innervation of hind limb muscles, profound defects at NMJs and a delay in the motor recovery compared with wild-type counterpart [60].

**Figure 1:**
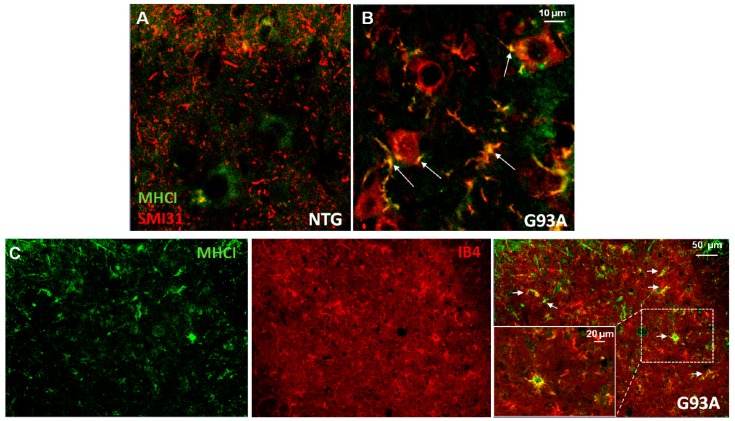
Major histocompatibility complex class I (MHCI) immunoreactivity in dystrophic neurites of motoneurons (MNs) and activated microglia. (**A**,**B**) Representative immunofluorescent microphotographs showing MHCI (green) and phosphorylated neurofilaments (SMI31) in L3–L4 ventral horn of normal control and SOD1G93A mice at the early phase of the disease. Note a diffuse cytosolic MHCI immunostaining in MNs of wild-type mice, while SMI31 appears only as punctiform staining of axons of different calibers in the spinal cord parenchyma (**A**). In SOD1G93A mice, SMI31 immunolabeling is prevalent in MN somata and in dystrophic neurites/axons around MNs and partially co-localizes with MHCI (white arrows—yellow). (**C**) Representative immunofluorescent microphotographs showing the colocalization of MHCI (green) and activated microglia (Ib4) in the ventral horn of SOD1G93A mice at the early phase of the disease (white arrows—yellow). Note in the magnification the presence of MHCI in reactive microglia.

In recent years, the role of MHCI has been thoughtfully investigated in SOD1^G93A^ mouse models [35,61]. We recently performed a detailed analysis of MHCI distribution in C57SOD1^G93A^ mice at the early phases of the disease before the onset of motor symptoms. We observed that, while MHCI is confined to lumbar spinal cord MN perikarya under basal conditions, MHCI distributes in synapse terminals (Figure 1A,B) and efferent motor axons in pathological conditions [61]. In particular, we observed a high accumulation of MHCI in peripheral motor axons and NMJs of C57SOD1^G93A^ mice during disease progression. This effect was associated with increased levels of the Lmp7 immunoproteasome subunit and β2 microglobulin (β2m), in both MN somata and peripheral axons. This indicates that motor axons may locally produce and expose MHCI “loaded” with antigenic peptides to be presented to cytotoxic T cells that infiltrate the PNS of C57SOD1^G93A^ mice [44]. Interestingly, we found that this process is much less activated in rapidly progressing 129SvSOD1^G93A^ mice that do show an earlier axonal impairment and NMJ denervation [61]. Consistently, SOD1^G93A^ mice, when crossed with β2m^−/−^ mice, showed an anticipation of motor dysfunction, which correlates with an earlier axonal impairment and denervation of hind limb muscles [62]. On the contrary, increasing the levels of MHCI specifically into MNs through the use of adeno-associated virus type 9 (AAV9) vectors, significantly delayed the disease progression of mice [35].

In the PNS, successful axonal regeneration depends on the coordinated effort of non-neuronal cells that, while motor axon debris is removed, release extracellular matrix molecules, cytokines, and growth factors that support axon regrowth [63]. In this context, CD8^+^ T cells infiltrating the sciatic nerves may be essential not only to promote the phagocytosis of myelin debris by macrophages but also to limit the activity of effector T cells and macrophages once the remyelination is completed (recovery phase) [64].

Why is the MHCI activated in the MNs of C57SOD1^G93A^ mice? As discussed above, proteinopathy and the neuroinflammation are key players in ALS. In fact, the accumulation in the MNs of misfolded proteins genetically modified (e.g., mSOD1) or oxidized, is most likely the *primum movens* in the cascade leading to MN degeneration. Usually, misfolded aberrant proteins activate a complex protein quality control system in cells that include the constitutive ubiquitin-proteasome pathway, the ERAD, and autophagy. 

We and other groups found that, while the ubiquitin-proteasome machinery is impaired in the MNs of mSOD1 mice, the immunoproteasome subunits Lmp7, Lmp2, and Lmp10 (responsible for the production of antigenic peptides through the MHC I pathway) become strongly activated [42]. This suggests that, in degenerating MNs, misfolded proteins may be degraded and fragments exposed on the cell membrane to promote the interaction with immune cells. This process may be activated by neuroinflammation triggered by the same misfolded proteins in the MNs via the release of danger-associated molecular pattern molecules (DAMPs), including ROS, HMGB1, and HSPs, which activate glial and immune cells to produce inflammatory cytokines, including TNFα and INFγ [65,66]. While this process may be relevant to the early phase of the disease, at the advanced disease stage, the massive reactive astrocytosis in association with the ER stress in ALS MNs might be responsible for the reduced expression of MHCI in the lumbar spinal MNs of SOD1^G93A^ mice and ALS patients [35] with the consequent loss of compensatory NMJ re-innervation. In fact, through in vitro studies on astrocytes/MNs co-cultures from SOD1^G93A^ mice, Song et al. [35] suggested that reactive astrocytes may secrete ER stress inducers that result in a loss of MHCI in MNs increasing their susceptibility to ALS astrocyte-induced toxicity. In support of this, increased MHCI expression specifically in MNs protected them from ALS astrocyte-induced toxicity. 

Although different studies have suggested the involvement of ER stress signaling in ALS [67,68,69], how ALS astrocytes cause ER stress in MNs is still unknown. It is also important to emphasize that other non-neuronal cells may also cause ER stress. In particular, microglia in the CNS monitor pathogen-associated molecular patterns (PAMPs) and DAMPs and may in turn become activated and secrete pro-inflammatory molecules that may affect MN homeostasis [70].

MHC class I complexes are assembled in the endoplasmic reticulum through the concerted action of multiple ER-resident proteins. In response to ER stress, cells induce a highly conserved cellular stress response called the unfolded protein response (UPR) in an attempt to maintain their homeostasis [71]. In order to maintain quality control, the cell also employs ERAD and attenuates translation of global mRNA to alleviate protein load within the lumen [72,73]. These systems are generally exploited by viral immunoevasins to interfere with antigen presentation pathways [74]. For example, the HCMV glycoprotein US11 interacts with the ERAD complex leading to dislocation of MHCI heavy chains from the ER membrane into the cytosol where they are degraded by the proteasome [74]. A protein associated with US11 and that directly interacts with MHCI heavy chains is a human homolog of yeast Der 1 p, known as Der-like protein 1 (Derlin-1). It is noteworthy that, in primary MNs, mSOD1 interacts with Derlin-1 to induce ER-stress, ASK1 activation, and MN death. Particularly, mSOD1 does trap ERAD substrates on the complex composed of SOD1mut–Derlin-1–VIMP and thereby inhibits the subsequent transfer of ERAD substrates to the proteasome [75]. Thus, astrocytes can exploit the same mechanism of the virus and secrete immunoevasin–like proteins to interfere with MHCI assembly into MNs and induce ER-stress in cooperation with mSOD1. 

## 3. The Expression of Major Histocompatibility Complex Class I (MHCI) by Non-Neuronal Cells 

In the previous section, we focused on the expression of MHCI by MNs in pathological conditions. However, other non-neuronal cell types surrounding MNs within CNS and PNS activate MHCI in different pathological conditions [44].

Bombeiro et al. [76] showed that astrocyte activation and reactivity in vitro are related to MHCI expression and astrogliosis could be downregulated by silencing MHCI mRNA synthesis. Silencing of β2m mRNA decreased expression of the astrocytic marker (GFAP—glial fibrillary acidic protein) as well as pro-inflammatory cytokines (TNF-α, IL-1, IL-6, IL-12, and IL-17). In this scenario, preserved synaptophysin immunolabeling in co-cultures of neurons and astrocytes was observed. These data define a role of MHCI in governing the state of activation of astrocytes in vitro. However, these cells are unable to activate MHCI in vivo. In fact, no activation was observed in the spinal cord of SOD1^G93A^ mice during the disease progression [44]. On the contrary, microglia surrounding MNs, in the spinal cord of ALS mice, expressed high levels of MHCI at disease onset [44] (Figure 1C). Increased MHCI expression by glial cells has been previously reported in the spinal cord of mice after viral infection [77,78] or sciatic nerve injury [49,79] and in the hippocampus of advanced aging rats [80]. At CNS level, microglia is the principal antigen presenting cell and one of the main culprits of the non-cell autonomous MN death in ALS [81,82].

Microglia purified from adult mice injected intracerebrally with OVA efficiently stimulate OVA-specific CD8^+^ T cells, thereby showing that microglia takes up and process exogenous antigen into MHC class I in vivo [83]. At the same time, efficiently stimulated adult microglia cross-prime naïve CD8^+^ T cells injected into the brain [84]. These observations offer new insights on the immune role of MHCI during inflammation. Activated microglia induce MHCI to promote recruitment and propagation of cytotoxic T cells so that targeting the inhibition of MHCI in these cells would result in the attenuation of pro-inflammatory processes after an insult or during the progression of the disease. 

At the peripheral level, we recently reported the activation of MHCI by Schwann cells following sciatic nerve injury [79] or during ALS disease onset [61]. In C57SOD1^G93A^ mice, the MHCI activation of SCs correlates with the specific infiltration of CD8^+^ T cells in sciatic nerves, while the activation of autophagocytosis by SCs and the recruitment of macrophages are related to the coordinate and specific activation of both CCL2 and complement C3 subunit [61]. In the crushed nerve, MHCI protein expression and CD8^+^ T lymphocytes infiltration displayed similar patterns, peaking at two weeks after lesion and decreasing thereafter [79]. In addition, two weeks after crushing, most of the MHCI immunolabeling was restricted to glial and infiltrating cells, especially to macrophages. 

Considering that CD8^+^ T cells are involved in antigen recognition via TCR and MHCI interaction and given the activation of the immunoproteasome in the sciatic nerves of SOD1^G93A^ mice, it seems reasonable that, in the nerve, MHCI greatly contributes to the inflammatory immune response following injury rather than to the axonal plasticity. 

However, the contribution of the inflammatory response in the PNS starkly contrasts with that of the CNS, where the activity of nearby cells (microglia, astrocytes, and infiltrating immune cells) exacerbates cell death and damage by releasing toxic pro-inflammatory mediators over an extended period of time [85]. Successful axonal regeneration depends on the coordinated efforts of non-neuronal cells to remove motor axon debris In fact, Bombeiro et al. [86] demonstrated that enhanced immune response in immunodeficient mice improves peripheral nerve regeneration following axotomy [86]. In addition, in SOD1^G93A^ mice, we showed that the increased expression of MHCI and CCL2 by motor axons and Schwann cells, and the subsequent infiltration of peripheral macrophages and CD8^+^ T cells correlates with a delayed muscle denervation [61]. These findings indicate that Schwann cells as well as cytotoxic T cells and peripheral macrophages recruited in the sciatic nerve may cooperate for the targeted destruction of defective motor fibers (expressing axon growth inhibitors, e.g., myelin debris) in an attempt to promote the dedifferentiation and proliferation of Schwann cells and create a growth-permissive milieu for new neurites generated in the presence of axonal injury.

## 4. Conclusions

**Figure 2:**
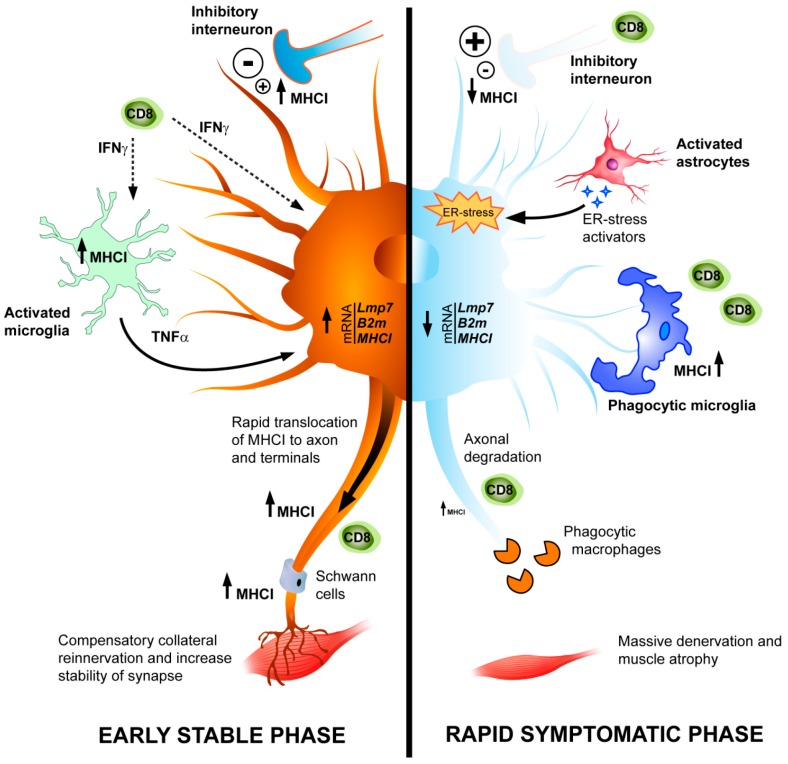
Schematic representation of dysregulation of MHCI in MNs during ALS pathology. Left side: in SOD1^G93A^ mice, at the early phase of the disease, a marked upregulation of mRNAs for MHCI and associated components, immunoproteasome (LMP7) and β2 microglobulin (β2m), occurs in MNs in response to accumulation of misfolded proteins (i.e., mutant SOD1) and presumably to the release of pro-inflammatory cytokines from activated microglia and astrocytes. Once transduced, MHCI is rapidly translocated to motor axons and terminals leaving the soma almost deprived of MHCI protein. This is associated with recruitment of T cells in sciatic nerves and axon terminals. Since the retraction of damaged motor axons at the neuromuscular junctions is the earliest event in ALS, we hypothesize that the upregulation of MHCI in the periphery may activate cytotoxic T cells to create a growth-permissive milieu, which promotes the pruning of damaged motor axons and the compensatory collateral reinnervation of muscles. In addition, MHCI maintains the proper activity of Schwann cells (SCs) in the motor axons and terminal Schwann cells (tSCs) at the neuromuscular junctions providing stability and synapse homeostasis. Right side: at the symptomatic phase, the highly reactive astrocytes around MNs contribute significantly to the reduction of MHCI through the activation of MHCI inhibitory receptor. A sustained immunoreactivity for MHCI persists in microglia with a phagocytic phenotype. The levels of MHCI, which is nearly absent in the MN perikarya, decrease also in motor axons and terminals. The skeletal muscles are massively denervated at this stage resulting in loss of function, atrophy, and motor paralysis. The activated microglia in the spinal cord overexpressing MHCI may increase the recruitment of CD8^+^ T cells contributing to the removal of damaged MNs in concert with macrophages at the final stage. ↑ upregulation; ↓ downregulation; Dashed line arrow—secretion of soluble factor; Long continuous arrow: effects of activated glia.

Increasing evidence indicates that mechanisms related to the adaptive immune response play an important role in ALS. MNs, when damaged by either an acute injury or chronic disease such as ALS, activate a series of molecules closely associated with the immune response. In this review, we focused on the potential role of MHCI in the progression of this devastating disease. The expression of MHCI in lesioned MNs has been reported to activate a series of compensatory mechanisms aimed to protect themselves from cell autonomous and non-cell-autonomous toxic mechanisms and to promote the regeneration of their axons that eventually leads to muscle reinnervation. Taking into account results hitherto obtained, we formulated a hypothesis for the role of MHCI during ALS progression, which is illustrated in Figure 2. During the early phase of the disease, MHCI molecules are synthesized by MNs in the lumbar spinal cord and rapidly transported to synapse terminals to limit the increased excitatory neurotransmission, an important pathogenic mechanism in ALS [87]. In fact, it has been recently reported an increase excitatory synaptic inputs and dendritic spine densities in the motor cortex of transgenic ALS mice long before an overt degenerative phenotype [88]. At the same time, MHCI subjected to anterograde axonal transport may promote a process of local immune response, which in synergy with Schwann cells phagocytes degraded material to allow the regeneration and collateral muscle reinnervation of peripheral motor axons [61]. As the disease progresses, increased astrocytosis leads to massive ER stress in MNs, which limits the exposition of MHCI on cell membranes of MNs and makes them more vulnerable to death. On the contrary, an increased expression of MHCI in microglia surrounding MNs may be detrimental through the recruitment of cytotoxic CD8^+^ T cells and the exacerbation of inflammation in the CNS. Thus, while increasing MHCI within MNs may be a useful strategy to counteract the denervation atrophy of muscles and delay disease progression, its activation in microglia could have a deleterious effect. 

Based on this information, the function of MHCI in the CNS may be cell-type-dependent. To date, we know that neuronal MHCI plays a crucial role during development and plasticity in CNS, whereas the role for glial MHCI deserves further analysis. Furthermore, it is likely that MHCI involves different interacting counterparts and signaling depending on the environmental stimulus. In the ALS context, further investigations, wherein models different from those characterized by mSOD1 mutations are used, will be necessary in the future. 

The failure of anti-inflammatory and anti-immune therapies in clinical trials [89] support a reconsideration of the role of the immune system in ALS. We expect that a better understanding of the molecular mechanisms of cross-talk between damaged MNs and the immune response in ALS mice should help in finding new approaches to promoting axonal regeneration and muscle reinnervation in ALS. A specific cell-targeted strategy aimed at modulating immune molecules such as MHC I is recommended for effective therapeutic response in ALS.
